# The correlation of circulating pro‐angiogenic miRNAs’ expressions with disease risk, clinicopathological features, and survival profiles in gastric cancer

**DOI:** 10.1002/cam4.1618

**Published:** 2018-07-12

**Authors:** Wei Peng, Ya‐Nan Liu, Si‐Qiang Zhu, Wen‐Qiang Li, Feng‐Cheng Guo

**Affiliations:** ^1^ Department of General Surgery Guangdong General Hospital Zhuhai Hospital (Zhuhai Golden Bay Center Hospital) Zhuhai China; ^2^ Deparment of General Surgery No. 211 Hospital of PLA Harbin China

**Keywords:** circulating, gastric cancer, miRNA, pro‐angiogenic, prognosis

## Abstract

This study aimed to explore the correlation of circulating pro‐angiogenic miRNAs’ expressions with risk, clinicopathological features, and survival profiles in gastric cancer (GC). Three hundred and thirty‐three GC patients underwent radical resection and 117 health controls (HCs) were recruited for this study. Plasma samples were obtained from GC patients before the operation and from HCs after enrollment. Fourteen pro‐angiogenic miRNAs were asseassed by quantitative polymerase chain reaction (qPCR). Disease‐free survival (DFS) and overall survival (OS) of GC patients were calculated and the median follow‐up duration was 36.0 months. Seven out of 14 pro‐angiogenic miRNAs including let‐7f, miR‐17‐5p, miR‐18a, miR‐19b‐1, miR‐20a, miR‐210, and miR‐296 were observed to be elevated in GC patients compared with HCs. MiR‐18a, miR‐20a, and miR‐210 disclosed good predictive values of GC risk. Six pro‐angiogenic miRNAs including miR‐17‐5p, miR‐92a, miR‐210, miR‐20a, miR‐18a, and miR‐296 expressions were positively while 1 pro‐angiogenic miRNA (miR‐130a) was negatively correlated with tumor malignancy degree in GC patients. K‐M curve disclosed that 5 pro‐angiogenic miRNAs including miR‐17‐5p, miR‐18a, miR‐20a, miR‐92a, and miR‐210 correlated with worse DFS, while 4 pro‐angiogenic miRNAs including miR‐17‐5p, miR‐18a, miR‐20a, and miR‐210 associated with shorter OS. Further multivariate Cox's analysis revealed that miR‐17‐5p, miR‐18a, miR‐20a, and miR‐210 were independent predictive factors for unfavorable DFS and OS. In conclusion, circulating pro‐angiogenic miRNAs could serve as novel noninvasive biomarkers for disease risk and malignancy degree, and miR‐17‐5p, miR‐18a, miR‐20a, and miR‐210 are independent factors predicting poor prognosis in GC patients.

## 1 INTRODUCTION

Gastric cancer (GC) is a complex heterogeneous disease, which ranks as the fifth most common cancer and the third cause of cancer deaths worldwide.^1^, ^2^ Higher incidence of GC is observed in China, Japan, Latin America, and Eastern Europe; in addition, China has considered GC as the commonest malignancy and the third leading country cause of death in 2010.^3^, ^4^, ^5^ GC mainly results from the interaction between host factors (including inherited or acquired factors) and the environment, with risk factors including *Helicobacter pylori* infection, smoking, alcohol, salt, and obesity.^6^, ^7^ Despite that many improvements have been realized for prolonging survival in GC patients such as optimized screening schedules, advanced surgical techniques, novel drugs, and individual treatment strategy, the prognosis of GC is still far more from satisfactory with 5‐year overall survival (OS) ranging from 12% to 98% according to the malignancy degree.^8^, ^9^ Therefore, exploration of novel and feasible biomarkers for disease‐risk prediction, progression monitoring, and long‐term prognosis is essential for the management of GC.

Blood vessels, which are lined with an inner layer of endothelial cells, form a dense network for supplying oxygen to all tissues of the body.^10^ It is identified by studies that angiogenesis would be deregulated in many diseases, and formation of new blood vessels supports disease development especially for various cancers.^11^ According to previous studies, the tumor growth in cancers such as GC, hepatocellular carcinoma, endometrial carcinoma, and renal cell carcinoma is angiogenesis‐dependent, thereby inhibition of angiogenesis during the processes of tumor growth is becoming a popular therapeutic strategy for cancers including GC. MicroRNAs (miRNAs), the small noncoding RNA molecules, regulate gene expression at the posttranscription level by binding to 3′ untranslated region (UTR) of the target gene.^12^, ^13^ Plenty of evidences have indicated that miRNAs play important roles in physiological and pathological processes in cancers, such as cell survival, proliferation, migration, and metastasis. For instance, miR‐155 expression is found to be upregulated in breast cancer compared with,^14^ and aberrant expression of miR‐196a is associated with cells apoptosis, invasion, and proliferation in pancreatic cancer.^15^ In addition, miRNA‐21 is identified as an oncogene in various malignant tumor tissues including GC.^16^ Considering pro‐angiogenic miRNAs have crucial effects on tumorigenesis and tumor progression, we hypothesized that they may also participate in the development and progression of GC, whereas few studies identify the diagnostic and prognostic values of multiple circulating pro‐angiogenic miRNAs in GC patients.

Thus, we conducted this study to investigate the correlation of circulating pro‐angiogenic miRNAs’ expressions with risk, clinicopathological features, and survival profiles of GC.

## 2 MATERIALS AND METHODS

### 2.1 Participants

Three hundred and thirty‐three GC patients underwent radical resection at Department of General Surgery in Guangdong General Hospital Zhuhai Hospital, Zhuhai Golden Bay Central Hospital between Jan 2012 and Dec 2015 and consecutively recruited for this study. The inclusion criteria were: (1) Diagnosed as primary GC according to clinical, imaging, and pathological findings; (2) Age above 18 years; (3) Classified as TNM stage I‐III by the 7th edition of the American Joint Committee on Cancer (AJCC) cancer staging manual; (4) Able to be followed up regularly; (5) About to undergo GC radical resection on demands. As with the diagnostic method, patients were clinically diagnosed as GC by enhanced CT or MRI, and subsequently confirmed by pathologic biopsy. Patients with the following conditions were excluded: (1) Received neo‐adjuvant therapies before operation; (2) History of other tumors or hematological malignance; (3) Accompanied with severe infection, severe kidney dysfunction, or severe hepatic dysfunction; (4) In pregnancy or lactation, or planning for pregnancy. In addition, 117 healthy controls (HCs) in the same duration were enrolled in this study, which were age and gender matched to GC patients.

### 2.2 Treatment and sample collection

All GC patients received radical resection on demand according to clinical practice, disease condition, and patients’ willingness. After the operation, patients underwent adjuvant chemotherapy, radiotherapy, chemoradiotherapy, or did not receive adjuvant therapies depending on the disease condition and clinical practice. In detail, 44 (13.2%) patients were pathologically diagnosed as lymph node‐negative, who did not receive adjuvant therapy; 214 (64.3%) patients received postoperative adjunctive therapy of ECF (eirubicin, cis‐platinum and 5‐FU) or single drug S‐1; 35 (10.5%) patients received postoperative radiotherapy; other 40 (12.0%) patients received radiotherapy (45‐50.4 Gy) combined with fluorouracil‐based radiotherapy sensitivity enhancing agent (5‐FU or capecitabine) plus sequential therapy of 5‐FU (with or without leucovorin) or capecitabine. Peripheral blood sample was obtained from each GC patient before the operation and from each HC after enrollment; subsequently, plasma was immediately separated and stored in liquid nitrogen for miRNAs’ detection.

### 2.3 qPCR detection for pro‐angiogenic miRNAs

Fourteen candidate pro‐angiogenic miRNAs were selected by analyzing a previous study.^17^ Total RNA was extracted from the plasma sample by Trizol LS kit (Takara, Japan). The reverse transcription of RNA was conducted by One Step Primer Scrip miRNA cDNA Synthesis Kit (Takara). qPCR procedures were conducted as follows: all reactions were incubated on a 96‐well plates at 95°C for 5 minutes, followed by 40 cycles of amplification, and each cycle included denaturation at 95°C for 5 seconds, annealing at 61°C for 15 seconds, and extension at 72°C for 30 seconds. U6 expression was used as the internal reference to normalize miRNA expressions, and the expressions of these candidate miRNAs were calculated by 2^−ΔΔct^ method. The primers used in this study were listed in Table 1.

**Table 1:** Primer information

Gene	Forward primer (5′‐>3′)	Reverse primer (5′‐>3′)
let‐7b	ACACTCCAGCTGGGTGAGGTAGTAGGTTGTGT	ACACTCCAGCTGGGACTGCAGTGAAGGCACTT
let‐7f	ACACTCCAGCTGGGTGAGGTAGTAGATTGTAT	ACACTCCAGCTGGGACTGCAGTGAAGGCACTT
miR‐17‐5p	ACACTCCAGCTGGGTGAGGTAGTAGGTTGTGT	ACACTCCAGCTGGGACTGCAGTGAAGGCACTT
miR‐17‐3p	ACACTCCAGCTGGGACTGCAGTGAAGGCACTT	ACACTCCAGCTGGGACTGCAGTGAAGGCACTT
miR‐18a	ACACTCCAGCTGGGTAAGGTGCATCTAGTGCA	ACACTCCAGCTGGGACTGCAGTGAAGGCACTT
miR‐19a	ACACTCCAGCTGGGAGTTTTGCATAGTTGCAC	ACACTCCAGCTGGGACTGCAGTGAAGGCACTT
miR‐19b‐1	ACACTCCAGCTGGGAGTTTTGCAGGTTTGCAT	ACACTCCAGCTGGGACTGCAGTGAAGGCACTT
miR‐20a	ACACTCCAGCTGGGTAAAGTGCTTATAGTGCA	ACACTCCAGCTGGGACTGCAGTGAAGGCACTT
miR‐92a	ACACTCCAGCTGGGTATTGCACTTGTCCCGGC	ACACTCCAGCTGGGACTGCAGTGAAGGCACTT
miR‐126	ACACTCCAGCTGGGCATTATTACTTTTGGTAC	ACACTCCAGCTGGGACTGCAGTGAAGGCACTT
miR‐130a	ACACTCCAGCTGGGTTCACATTGTGCTACTGT	ACACTCCAGCTGGGACTGCAGTGAAGGCACTT
miR‐210	ACACTCCAGCTGGGAGCCCCTGCCCACCGCAC	ACACTCCAGCTGGGACTGCAGTGAAGGCACTT
miR‐296	ACACTCCAGCTGGGAGGGCCCCCCCTCAATCC	ACACTCCAGCTGGGACTGCAGTGAAGGCACTT
miR‐378	ACACTCCAGCTGGGCTCCTGACTCCAGGTCCT	ACACTCCAGCTGGGACTGCAGTGAAGGCACTT
U6	CTCGCTTCGGCAGCACA	AACGCTTCACGAATTTGCGT

### 2.4 Data collection

In GC patients, age, gender, nationality, history of familial cancer, history of smoke, history of drink, *H. pylori* Infection, adjuvant chemotherapy, adjuvant radiotherapy, tumor location, pathological grade, tumor size, and TNM stage were recorded. In HCs, age and gender information was collected.

### 2.5 Follow‐up

Gastric cancer patients were followed up every 1‐3 months in the first year, and every 6‐12 months in the following duration. The median follow‐up duration was 36.0 months (1/4‐3/4 quartile: 26.0‐44.5 months, range 3.0‐59.0 months) and the last follow‐up month was 31 Dec 2017. Disease‐free survival (DFS) was calculated from the date of operation to the time of relapse or death of any cause, while overall survival (OS) was calculated from the date of operation to the time of death of any cause.

### 2.6 Statistics

Statistical analysis was performed using SPSS 22.0 software (IBM, USA) and Graphpad Prism 6 software (GraphPad Software Inc, USA). Data were presented as mean ± SD, count (%), or median (1/4‐3/4 quartile). Each miRNA was classified as high expression and low expression according to its median value. Comparison between 2 groups was determined by Wilcoxon rank sum test. Correlation was analyzed by Spearman test. ROC curves were performed to evaluate the predictive values of miRNAs for GC risk. Kaplan‐Meier (K‐M) curves and log‐rank test were used to evaluate DFS and OS. Univariate Cox's proportional hazard regression was applied to assess factors affecting DFS and OS. Multivariate Cox's proportional hazard regression was further conducted to evaluate independent predictive factors for DFS and OS. *P* value <.05 was considered significant.

## 3 RESULTS

### 3.1 Baseline characteristics

Mean age of GC patients (N = 333) and HCs (N = 117) was 59.42 ± 11.06 years and 57.95 ± 13.40 years, respectively. There were 179 males and 154 females in GC patients, while 66 males and 51 females in HCs. No difference of age (*P *=* *.243) or gender (*P *=* *.620) was observed between GC patients and HCs. As for GC patients, the number of cases with tumor location at cardia, gastric body, and gastric antrum was 81 (24.3%), 39 (11.7%), and 213 (64.0%), and mean tumor size was 3.15 ± 1.24 cm. According to pathological grade, there were 50 (15.0%), 238 (71.5%), and 45 (13.5%) patients classified into G1, G2, and G3 respectively. In addition, 44 (13.2%), 142 (42.6%), and 147 (44.1%) patients were at TNM stage I, II, and III, respectively. The other detailed features of GC patients were listed in Table 2.

**Table 2:** GC Patients’ characteristics

Parameters	GC patients (n%) (N = 333)
Age (y)	59.42 ± 11.06
Gender	
Male	179 (53.8)
Female	154 (46.2)
Nationality	
Han	322 (96.7)
Others	11 (3.3)
History of familial cancer	46 (13.8)
History of smoke	147 (44.1)
History of drink	129 (38.7)
*H. pylori* Infection	
Positive	124 (37.2)
Negative	209 (62.8)
Adjuvant chemotherapy	214 (64.3)
Adjuvant radiotherapy	35 (10.5)
Tumor location	
Cardia	81 (24.3)
Gastric body	39 (11.7)
Gastric antrum	213 (64.0)
Pathological grade	
G1	50 (15.0)
G2	238 (71.5)
G3	45 (13.5)
Tumor size (cm)	3.15 ± 1.24
T stage	
T1	9 (2.7)
T2	35 (10.5)
T3	284 (85.3)
T4	5 (1.5)
N stage	
N0	114 (34.2)
N1	77 (23.1)
N2	123 (36.9)
N3	19 (5.7)
M stage	
M0	333 (100.0)
M1	0 (0.0)
TNM stage	
I	44 (13.2)
II	142 (42.6)
III	147 (44.1)

GC, gastric cancer.

Data were presented as mean ± SD, count (%), or median (1/4‐3/4 quartile).

### 3.2 Comparison of miRNAs between GC patients and HCs

**Figure 1:**
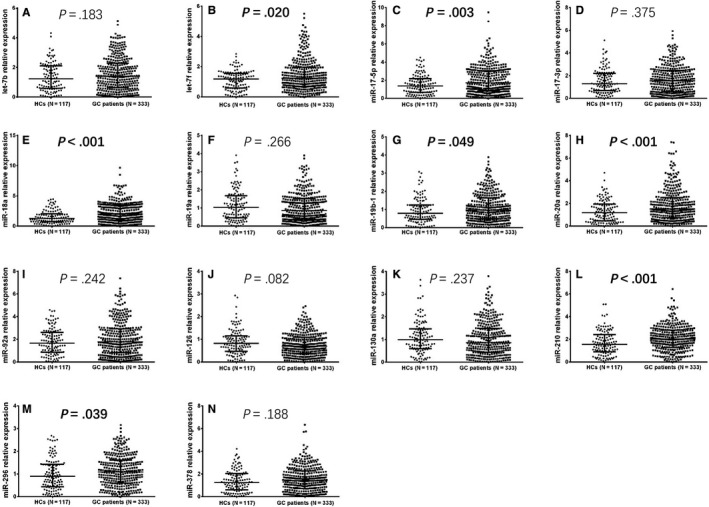
Comparison of candidate miRNAs’ relative expressions between GC patients and HCs. Expressions of let‐7f (B), miR‐17‐5p (C), miR‐18a (E), miR‐19b‐1 (G), miR‐20a (H), miR‐210 (L), and miR‐296 (M) were elevated in GC patients compared with HCs, and miR‐126 expression was numerically higher in GC patients compared with HCs, but without significant difference (J). No difference in other miRNAs’ expressions including let‐7b (A), miR‐17‐3p (D), miR‐19a (F), miR‐92a (I), miR‐130a (K), and miR‐378 (N) was found between GC patients and HCs. Comparison of candidate miRNAs’ expressions between GC patients and HCs were determined by Wilcoxon rank sum test. *P* value <.05 was considered significant

As shown in Figure 1, the expressions of plasma let‐7f (Figure 1B, *P* = .020), miR‐17‐5p (Figure 1C, *P* = .003), miR‐18a (Figure 1E, *P* < .001), miR‐19b‐1 (Figure 1G, *P* = .049), miR‐20a (Figure 1H, *P* < .001), miR‐210 (Figure 1L, *P* < .001), and miR‐296 (Figure 1M, *P* = .039) were higher in GC patients compared with HCs, and miR‐126 expression was numerically higher in GC patients compared to HCs, but without significant difference (Figure 1J, *P* = .082). No difference in other miRNAs’ expressions including let‐7b (Figure 1A, *P* = .183), miR‐17‐3p (Figure 1D, *P* = .375), miR‐19a (Figure 1F, *P* = .266), miR‐92a (Figure 1I, *P* = .242), miR‐130a (Figure 1K, *P* = .237), and miR‐378 (Figure 1N, *P* = .188) was found between GC patients and HCs.

#### 3.2.1 Candidate pro‐angiogenic miRNAs’ levels in distinguishing GC patients from HCs

**Figure 2:**
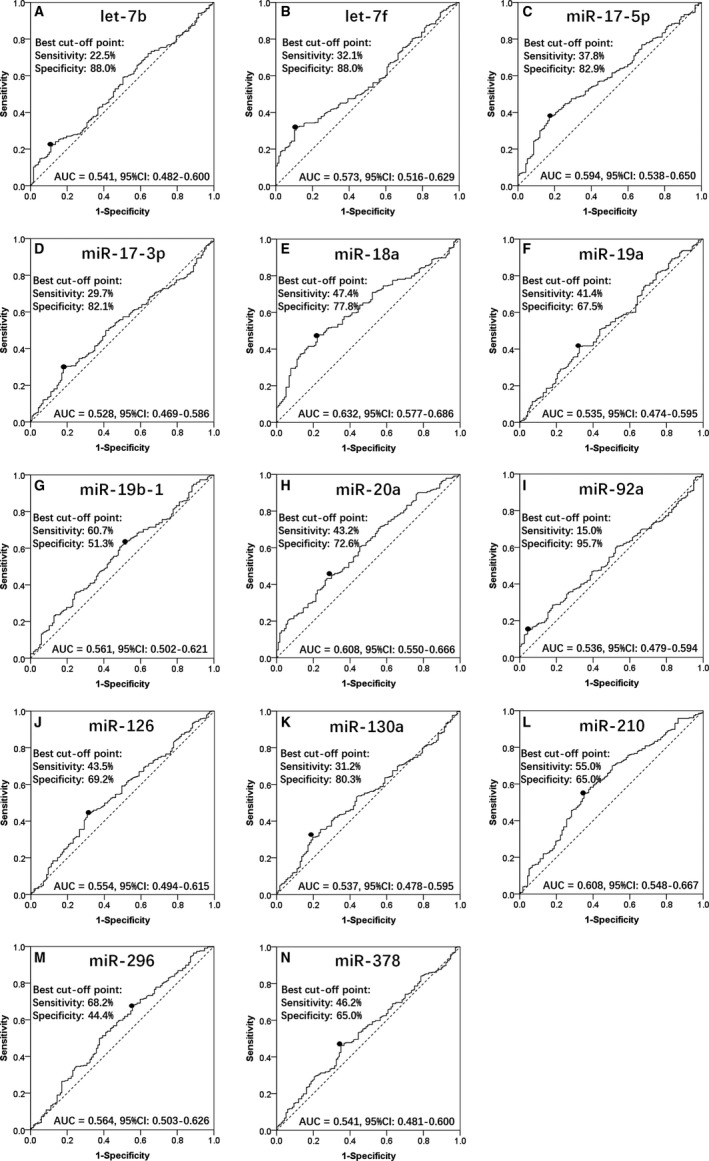
ROC curves were used to investigate the value of miRNAs in predicting GC risk. MiR‐18a (AUC: 0.632, 95% CI: 0.577‐0.686) (E), miR‐20a (AUC: 0.608, 95% CI: 0.550‐0.666) (H), and miR‐210 (AUC: 0.608, 95% CI: 0.548‐0.667) (L) disclosed good predictive values of GC risk. The sensitivity and specificity in miR‐18a, miR‐20a, and miR‐210 were 47.4% and 77.8%, 43.2% and 72.6%, and 55.0% and 65.0%, respectively. Other candidate miRNAs, including let‐7b (A), let‐7f (B), miR‐17‐5p (C), miR‐17‐3p (D), miR‐19a (F), miR‐19b‐1 (G), miR‐92a (I), miR‐126 (J), miR‐130a (K), miR‐296 (M), and miR‐378 (N), could not distinguish GC from HCs

ROC curves were drawn to investigate the value of miRNAs in predicting GC. As shown in Figure 2, miR‐18a (AUC: 0.632, 95% CI: 0.577‐0.686; (Figure 2E), miR‐20a (AUC: 0.608, 95% CI: 0.550‐0.666; Figure 2H), and miR‐210 (AUC: 0.608, 95% CI: 0.548‐0.667; Figure 2L) disclosed good predictive values of GC risk. The sensitivity and specificity in miR‐18a, miR‐20a, and miR‐210 were 47.4% and 77.8%, 43.2% and 72.6%, and 55.0% and 65.0%, respectively, at the best cut‐off point (the point where the sum of sensitivity and specificity is the largest). Other candidate miRNAs, including let‐7b (AUC: 0.541, 95% CI: 0.482‐0.600; Figure 2A), let‐7f (AUC: 0.573, 95% CI: 0.516‐0.629; Figure 2B), miR‐17‐5p (AUC: 0.594, 95% CI: 0.538‐0.650; Figure 2C), miR‐17‐3p (AUC: 0.528, 95% CI: 0.469‐0.586; Figure 2D), miR‐19a (AUC: 0.535, 95% CI:0.474‐0.595; Figure 2F), miR‐19b‐1 (AUC: 0.561, 95% CI: 0.502‐0.621; Figure 2G), miR‐92a (AUC: 0.536, 95% CI: 0.479‐0.594; Figure 2I), miR‐126 (AUC: 0.554, 95% CI: 0.494‐0.615; Figure 2J), miR‐130a (AUC: 0.537, 95% CI:0.478‐0.595; Figure 2K), miR‐296 (AUC: 0.564, 95% CI: 0.503‐0.626; Figure 2M), and miR‐378 (AUC: 0.541, 95% CI: 0.481‐0.600; Figure 2N) could not distinguish GC from HCs.

### 3.3 Correlation of candidate pro‐angiogenic miRNAs with key clinicopathological features in GC patients

The correlation of pro‐angiogenic miRNAs with clinicopathological features was exhibited in Table 3. MiR‐17‐5p was positively correlated with pathological grade (*R* = 0.212, *P *=* *.000), N stage (*R* = 0.151, *P *=* *.006), and TNM stage (*R* = 0.148, *P *=* *.007); miR‐18a was positively correlated with T stage (*R* = 0.157, *P *=* *.004), N stage (*R* = 0.143, *P *=* *.009), and TNM stage (*R* = 0.189, *P *=* *.001); miR‐20a was positively correlated with tumor size (*R* = 0.132, *P *=* *.016); miR‐92a was positively correlated with pathological grade (*R* = 0.181, *P *=* *.001); miR‐210 was positively correlated with pathological grade (*R* = 0.131, *P *=* *.017); miR‐296 was positively associated with N stage (*R* = 0.116, *P *=* *.034), while miR‐130a was negatively correlated with pathological grade (*R* = −0.151, *P *=* *.006).

**Table 3:** Correlation of candidate pro‐angiogenic miRNAs with key clinicopathological features in GC patients

Parameters	Pathological grade	Tumor size	T stage	N stage	TNM stage
let‐7b					
*R*	−0.041	−0.058	−0.024	0.016	−0.023
*P* value	.460	.291	.657	.778	.676
let‐7f					
*R*	0.046	0.003	0.046	0.075	0.058
*P* value	.402	.954	.406	.170	.294
miR‐17‐5p					
*R*	0.212	−0.018	0.096	0.151	0.148
*P* value	**.000**	.750	.080	**.006**	**.007**
miR‐17‐3p					
*R*	0.017	0.004	0.033	−0.029	−0.020
*P* value	.755	.944	.547	.594	.710
miR‐18a					
*R*	0.057	0.041	0.157	0.143	0.189
*P* value	.301	.459	**.004**	**.009**	**.001**
miR‐19a					
*R*	0.005	0.048	−0.089	−0.093	−0.094
*P* value	.933	.379	.106	.091	.087
miR‐19b‐1					
*R*	0.006	−0.105	−0.025	−0.003	−0.040
*P* value	.913	.055	.652	.960	.469
miR‐20a					
*R*	0.028	0.132	−0.030	−0.051	−0.014
*P* value	.617	**.016**	.588	.357	.803
miR‐92a					
*R*	0.181	−0.024	0.025	0.055	0.013
*P* value	**.001**	.661	.646	.314	.809
miR‐126					
*R*	−0.090	−0.016	−0.012	−0.013	−0.024
*P* value	.099	.765	.821	.815	.658
miR‐130a					
*R*	−0.151	−0.042	−0.064	−0.069	−0.072
*P* value	**.006**	.441	.241	.212	.188
miR‐210					
*R*	0.131	0.070	0.091	0.074	0.077
*P* value	**.017**	.202	.097	.180	.160
miR‐296					
*R*	0.084	−0.012	0.073	0.116	0.088
*P* value	.124	.831	.182	**.034**	.108
miR‐378					
R	0.061	0.018	−0.005	0.048	0.054
*P* value	.268	.748	.922	.380	.322

GC, gastric cancer.

Data were presented as *R* and *P* values. Correlation was analyzed by Spearman test. *P *<* *.05 was considered significant and highlighted in bold.

#### 3.3.1 Correlations of miRNAs’ expressions with adjuvant treatments

Distribution of adjuvant therapies including chemotherapy, radiotherapy, and chemoradiotherapy in the high and low groups of each miRNA was displayed in Table 4. MiR‐378 high expression was negatively correlated with adjuvant chemotherapy (*P* = .047). No other correlation of miRNAs’ expressions with adjuvant treatments was observed.

**Table 4:** Distribution of adjuvant treatments in the high and low groups of each miRNA

miRNAs	Adjuvant chemotherapy (N = 214)	Adjuvant radiotherapy (N = 35)	Adjuvant chemoradiotherapy (N = 40)	NO adjuvant therapy (N = 44)
let‐7b				
High	106 (49.5)	20 (57.1)	22 (55.0)	25 (56.8)
Low	108 (50.5)	15 (42.9)	18 (45.0)	19 (43.2)
*P* value	.877	.362	.487	.321
let‐7f				
High	108 (50.5)	19 (54.3)	21 (52.5)	21 (47.7)
Low	106 (49.5)	16 (45.7)	19 (47.5)	23 (52.3)
*P* value	.763	.579	.721	.762
miR‐17‐5p				
High	107 (50.5)	20 (57.1)	21 (52.5)	21 (47.7)
Low	107 (50.5)	15 (42.9)	19 (47.5)	23 (52.3)
*P* value	.941	.362	.721	.762
miR‐17‐3p				
High	111 (51.9)	20 (57.1)	24 (60.0)	22 (50.0)
Low	103 (48.1)	15 (42.9)	16 (40.0)	22 (50.0)
*P* value	.323	.362	.171	.983
miR‐18a				
High	112 (52.3)	18 (51.4)	21 (52.5)	17 (38.6)
Low	102 (47.7)	17 (48.6)	19 (47.5)	27 (61.4)
*P* value	.224	.843	.721	.110
miR‐19a				
High	103 (48.1)	16 (45.7)	17 (42.5)	25 (56.8)
Low	111 (51.9)	19 (54.3)	23 (57.5)	19 (43.2)
*P* value	.400	.605	.322	.321
miR‐19b‐1				
High	112 (52.3)	17 (48.6)	21 (52.5)	20 (45.5)
Low	102 (47.7)	18 (51.4)	19 (47.5)	24 (54.5)
*P* value	.224	.873	.721	.531
miR‐20a				
High	101 (47.2)	17 (48.6)	20 (50.0)	26 (59.1)
Low	113 (52.8)	18 (51.4)	20 (50.0)	18 (40.9)
*P* value	.194	.873	.984	.188
miR‐92a				
High	103 (48.1)	20 (57.1)	22 (55.0)	22 (50.0)
Low	111 (51.9)	15 (42.9)	18 (45.0)	22 (50.0)
*P* value	.400	.362	.487	.983
miR‐126				
High	104 (48.6)	18 (51.4)	20 (50.0)	22 (50.0)
Low	110 (51.4)	17 (48.6)	20 (50.0)	22 (50.0)
*P* value	.540	.843	.984	.983
miR‐130a				
High	106 (49.5)	18 (51.4)	20 (50.0)	24 (54.5)
Low	108 (50.5)	17 (48.6)	20 (50.0)	20 (45.5)
*P* value	.877	.843	.984	.504
miR‐210				
High	111 (51.9)	14 (40.0)	17 (42.5)	20 (45.5)
Low	103 (48.1)	21 (60.0)	23 (57.5)	24 (54.5)
*P* value	.323	.218	.322	.531
miR‐296				
High	112 (52.3)	19 (54.3)	23 (57.5)	17 (38.6)
Low	102 (47.7)	16 (45.7)	17 (42.5)	27 (61.4)
*P* value	.224	.579	.302	.110
miR‐378				
High	98 (45.8)	16 (45.7)	18 (45.0)	23 (52.3)
Low	116 (54.2)	19 (54.3)	22 (55.0)	21 (47.7)
*P* value	**.047**	.605	.513	.730

Data were presented as count (%). Comparison was determined by Chi‐square test. *P* value <.05 was considered significant and highlighted in bold.

### 3.4 Survival profiles in GC patients

**Figure 3:**
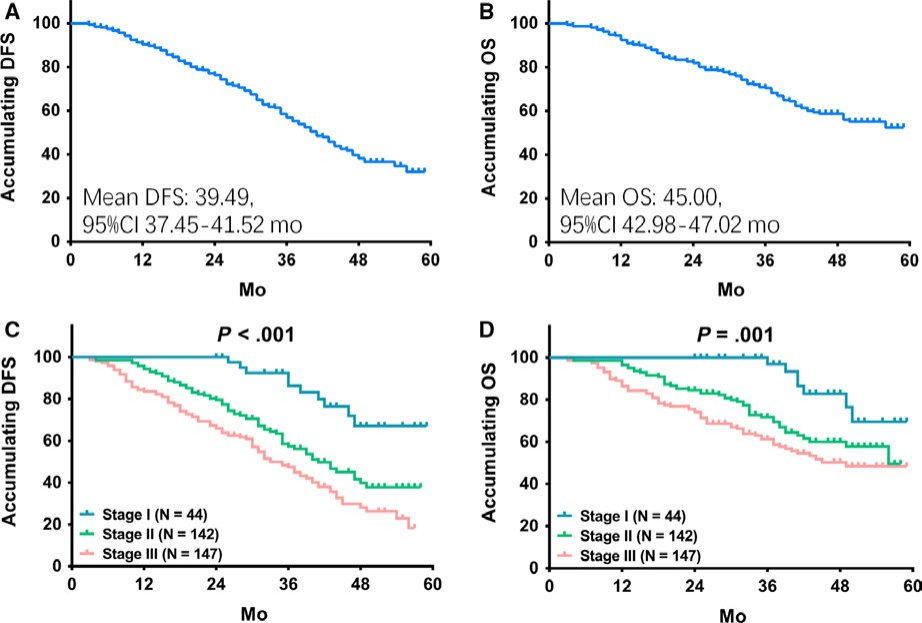
DFS and OS in GC patients. Mean value of DFS in GC patients was 39.49 (95% CI 37.45‐41.52) months (A), and mean value of OS in GC patients was 45.00 (95% CI 42.98‐47.02) months (B). K‐M curves were conducted to exhibit DFS and OS in GC patients

Kaplan‐Meier curves were conducted to exhibit DFS and OS in GC patients. As presented in Figure 3, mean value of DFS in GC patients was 39.49 (95% CI 37.45‐41.52) months (Figure 3A), and mean value of OS in GC patients was 45.00 (95% CI 42.98‐47.02) months (Figure 3B). As with survival profiles in each stage GC patients, higher stage of GC was correlated with shorter DFS (Figure 3C) as well as worse OS (Figure 3D).

### 3.5 Correlation of candidate miRNAs’ expressions with accumulating DFS

**Figure 4:**
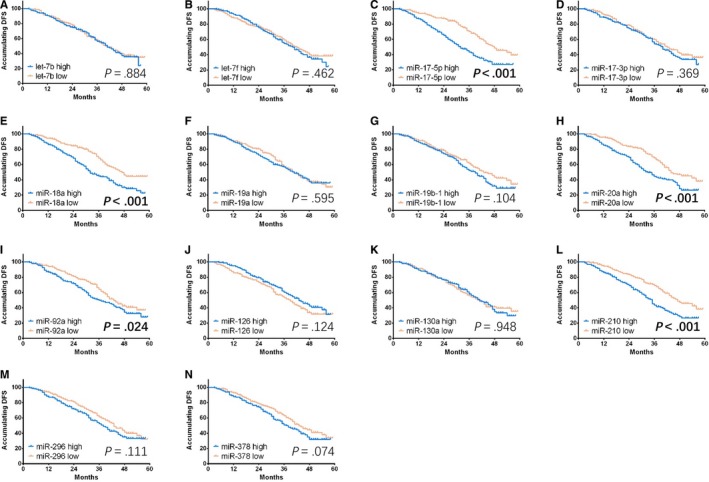
Comparison of DFS between GC patients with miRNAs’ high expression and miRNAs’ low expression. Elevated expressions of miR‐17‐5p (C), miR‐18a (E), miR‐20a (H), miR‐92a (I), and miR‐210 (L) were associated with shorter DFS compared with low expressions of the corresponding miRNAs. Furthermore, miR‐378 high expression was numerically correlated with decreased DFS, but without significant difference (N). No difference in correlation of DFS with other miRNAs between groups was observed. K‐M curves were used to exhibit DFS, and Log‐Rank test was conducted to compare DFS between groups. *P *<* *.05 was considered significant

High expressions of miR‐17‐5p (Figure 4C, *P* < .001), miR‐18a (Figure 4E, *P* < .001), miR‐20a (Figure 4H, *P* < .001), miR‐92a (Figure 4I, *P* = .024), and miR‐210 (Figure 4L, *P* < .001) were associated with shorter DFS compared with low expressions of these corresponding miRNAs. In addition, miR‐378 high expression was numerically correlated with decreased DFS, but without significant difference (Figure 4N, *P* = .074). No correlation of DFS with other miRNAs’ expressions was observed.

### 3.6 Analysis of factors affecting DFS

Univariate Cox's proportional hazard analysis revealed that miR‐17‐5p high expression (*P *<* *.001), miR‐18a high expression (*P *<* *.001), miR‐20a high expression (*P *<* *.001), miR‐92a high expression (*P *=* *.026), and miR‐210 high expression (*P *<* *.001) were associated with unfavorable DFS in GC patients (Table 5). Besides, higher pathological grade (*P *<* *.001), higher T stage (*P *<* *.001), higher N stage (*P *<* *.001), and higher TNM stage (*P *<* *.001) were correlated with decreased DFS in GC patients as well. In subsequent multivariate Cox's proportional hazard analysis, miR‐17‐5p high expression (*P *<* *.001), miR‐18a high expression (*P *<* *.001), miR‐19b‐1 high expression (*P *=* *.045), miR‐20a high expression (*P *=* *.003), and miR‐210 high expression (*P *=* *.015) were independent predictive factors for worse DFS; meanwhile, higher pathological grade (*P *<* *.001) and higher N stage (*P *=* *.013) were independent predictive factors for reduced DFS in GC patients as well.

**Table 5:** Analysis of factors affecting accumulating DFS

Parameters	Univariate Cox's proportional hazard regression				Multivariate Cox's proportional hazard regression			
	*P* value	HR	95% CI		*P* value	HR	95% CI	
			Lower	Higher			Lower	Higher
let‐7b high	.845	1.030	0.764	1.390	.957	0.991	0.711	1.382
let‐7f high	.466	1.118	0.828	1.508	.447	0.880	0.633	1.224
miR‐17‐5p high	**<.001**	2.145	1.577	2.917	**<.001**	1.930	1.383	2.694
miR‐17‐3p high	.374	1.146	0.849	1.546	.112	1.307	0.939	1.819
miR‐18a high	**<.001**	1.893	1.395	2.569	**<.001**	1.876	1.345	2.616
miR‐19a high	.598	1.084	0.804	1.462	.964	0.993	0.716	1.377
miR‐19b‐1 high	.108	1.280	0.947	1.729	**.045**	1.421	1.008	2.004
miR‐20a high	**<.001**	1.820	1.343	2.466	**.003**	1.679	1.190	2.368
miR‐92a high	**.026**	1.406	1.041	1.898	.073	1.353	0.972	1.885
miR‐126 high	.128	0.792	0.587	1.070	.513	0.893	0.636	1.254
miR‐130a high	.949	1.010	0.749	1.362	.056	1.376	0.992	1.907
miR‐210 high	**<.001**	1.848	1.363	2.505	**.015**	1.531	1.085	2.160
miR‐296 high	.115	1.272	0.943	1.717	.391	1.152	0.833	1.593
miR‐378 high	.077	1.311	0.971	1.770	.902	1.020	0.740	1.407
Age > 60 y	.673	0.937	0.694	1.266	.821	1.041	0.734	1.477
Gender (male)	.234	1.202	0.888	1.626	.730	0.938	0.654	1.346
Nationality (Han)	.926	0.962	0.426	2.174	.454	0.709	0.289	1.741
History of familial cancer	.635	1.100	0.741	1.633	.509	1.153	0.756	1.758
History of smoke	.460	0.892	0.659	1.208	.494	1.144	0.779	1.680
History of drink	.556	0.911	0.667	1.244	.122	0.732	0.493	1.087
*H. pylori* Infection positive	.320	1.169	0.859	1.590	.350	1.173	0.840	1.637
Adjuvant chemotherapy	.484	1.121	0.814	1.545	.352	0.837	0.574	1.218
Adjuvant radiotherapy	.973	1.008	0.625	1.625	.275	0.745	0.440	1.263
Tumor location (antrum)	.558	1.097	0.805	1.495	.598	1.096	0.780	1.539
Pathological grade	**<.001**	1.822	1.379	2.406	**<.001**	2.394	1.598	3.585
Tumor size > 3 cm	.107	1.282	0.948	1.734	.965	1.009	0.691	1.473
T stage	**<.001**	2.005	1.371	2.931	.219	1.545	0.772	3.094
N stage	**<.001**	1.433	1.229	1.671	**.013**	2.541	1.217	5.309
TNM stage	**<.001**	1.786	1.416	2.254	.147	0.734	0.484	1.115

DFS, disease‐free survival.

Data were presented as *P* value, HR (hazard ratio), and 95% CI (confidence interval). Univariate and multivariate Cox's proportional hazard regressions were performed to analyze factors affecting accumulating DFS. *P *<* *.05 was considered significant and highlighted in bold. Pathological grade was scored as G1 = 1, G2 = 2, G3 = 3; T stage was scored as T1 = 1, T2 = 2, T3 = 3, T4 = 4; N stage was scored as N0 = 0, N1 = 1, N2 = 2, N3 = 3; TNM stage was scored as I = 1, II = 2, III = 3. The statistical analysis was carried out based on these definitions.

### 3.7 Correlation of candidate miRNAs’ expressions with accumulating OS

**Figure 5:**
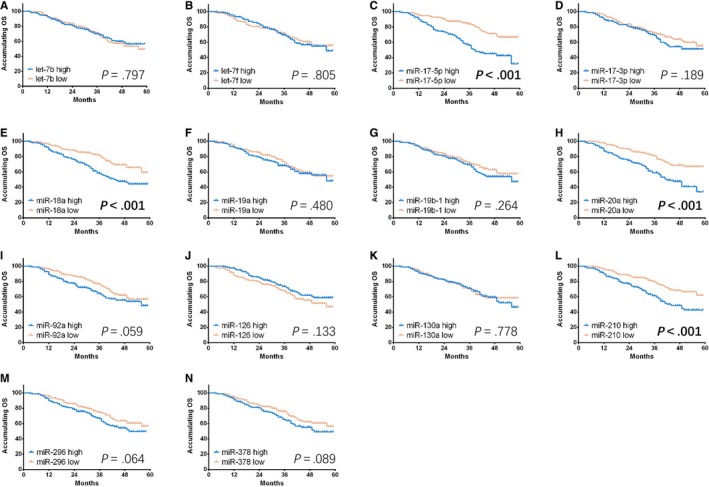
Comparison of OS between patients with miRNAs’ high expression and miRNAs’ low expression. OS in GC patients with high expressions of miR‐17‐5p (C), miR‐18a (E), miR‐20a (H), and miR‐210 (L) was shorter than that in patients with corresponding miRNAs’ low expressions. In addition, there was a decreasing trend of OS in patients with miR‐92a high expression (I), miR‐296 high expression (M), and miR‐378 high expression (N) compared to patients with corresponding miRNA low expressions, while no significant difference was found. No difference was found in correlation of OS between other miRNAs’ high expressions and low expressions. K‐M curves were applied to exhibit OS, and Log‐Rank test was conducted to compare OS between groups. *P *<* *.05 was considered significant

The OS in GC patients with high expressions of miR‐17‐5p (Figure 5C, *P* < .001), miR‐18a (Figure 5E, *P* < .001), miR‐20a (Figure 5H, *P* < .001), and miR‐210 (Figure 5L, *P* < .001) was shorter than that in patients with low expressions of these corresponding miRNAs. Moreover, there was a descending trend of OS in patients with high expression of miR‐92a (Figure 5I, *P* = .059), miR‐296 (Figure 5M, *P* = .064) as well as miR‐378 (Figure 5N, *P* = .089) compared to patients with low expressions of these corresponding miRNA, while no significant difference was found. No correlation of OS with other miRNAs’ expressions was found.

### 3.8 Analysis of factors affecting OS

According to univariate Cox's proportional hazard regression analysis, miR‐17‐5p high expression (*P *<* *.001), miR‐18a high expression (*P *<* *.001), miR‐20a high expression (*P *<* *.001), and miR‐210 high expression (*P *<* *.001) were associated with shorter OS in GC patients (Table 6). Additionally, higher pathological grade (*P *<* *.001), tumor size >3 cm (*P *=* *.027), higher T stage (*P *=* *.009), higher N stage (*P *<* *.001), and higher TNM stage (*P *<* *.001) were correlated with worse OS in GC patients. Further multivariate Cox's proportional hazard regression revealed that miR‐17‐5p high expression (*P *<* *.001), miR‐18a high expression (*P *=* *.001), miR‐20a high expression (*P *=* *.001), and miR‐210 high expression (*P *=* *.024) were independent predictive factors for reduced OS in GC patients, as well as higher pathological grade (*P *<* *.001) and higher N stage (*P *=* *.030). In addition, the summary of the expressions, diagnostic value, and prognostic value of miRNAs were exhibited in Table S1.

**Table 6:** Analysis of factors affecting accumulating OS

Parameters	Univariate Cox's proportional hazard regression				Multivariate Cox's proportional hazard regression			
	*P* value	HR	95% CI		*P* value	HR	95% CI	
			Lower	Higher			Lower	Higher
let‐7b high	.798	0.954	0.668	1.364	.461	0.859	0.574	1.286
let‐7f high	.806	1.046	0.732	1.494	.268	0.798	0.535	1.190
miR‐17‐5p high	**<.001**	2.666	1.828	3.890	**<.001**	2.308	1.537	3.468
miR‐17‐3p high	.193	1.269	0.886	1.816	.210	1.294	0.865	1.935
miR‐18a high	**<.001**	2.043	1.413	2.954	**.001**	1.977	1.312	2.979
miR‐19a high	.483	1.136	0.795	1.623	.907	0.977	0.660	1.447
miR‐19b‐1 high	.268	1.224	0.856	1.751	.204	1.306	0.865	1.971
miR‐20a high	**<.001**	2.272	1.569	3.290	**.001**	2.067	1.356	3.151
miR‐92a high	.062	1.406	0.983	2.013	.182	1.309	0.882	1.944
miR‐126 high	.137	0.762	0.533	1.090	.814	0.953	0.639	1.421
miR‐130a high	.780	1.052	0.737	1.503	.058	1.493	0.986	2.262
miR‐210 high	**<.001**	2.024	1.401	2.923	**.024**	1.577	1.062	2.342
miR‐296 high	.067	1.399	0.977	2.004	.252	1.263	0.847	1.883
miR‐378 high	.092	1.361	0.951	1.948	.561	1.121	0.763	1.647
Age > 60 y	.469	1.141	0.798	1.630	.089	1.443	0.946	2.201
Gender (male)	.205	1.264	0.880	1.815	.729	0.927	0.602	1.426
Nationality (Han)	.624	0.799	0.326	1.958	.296	0.578	0.207	1.617
History of familial cancer	.353	0.779	0.460	1.319	.598	0.859	0.488	1.511
History of smoke	.644	0.919	0.641	1.317	.543	1.150	0.733	1.805
History of drink	.287	0.815	0.559	1.188	.052	0.628	0.393	1.005
*H. pylori* Infection positive	.180	1.282	0.892	1.844	.484	0.841	0.518	1.365
Adjuvant chemotherapy	.746	0.940	0.648	1.365	.496	0.861	0.559	1.325
Adjuvant radiotherapy	.954	0.983	0.553	1.748	.417	0.772	0.413	1.443
Tumor location (antrum)	.244	1.251	0.858	1.824	.520	1.145	0.757	1.732
Pathological grade	**<.001**	2.276	1.626	3.187	**<.001**	3.247	1.964	5.369
Tumor size > 3 cm	**.027**	1.497	1.048	2.140	.137	1.394	0.900	2.159
T stage	**.009**	1.866	1.172	2.970	.105	2.016	0.864	4.704
N stage	**<.001**	1.422	1.181	1.711	**.030**	1.551	1.043	2.304
TNM stage	**<.001**	1.676	1.270	2.212	.215	1.727	0.729	4.090

OS, overall survival.

Data were presented as *P* value, HR (hazard ratio), and 95% CI (confidence interval). Univariate and multivariate Cox's proportional hazard regressions were performed to analyze factors affecting accumulating OS. *P *<* *.05 was considered significant and highlighted in bold. Pathological grade was scored as G1 = 1, G2 = 2, G3 = 3; T stage was scored as T1 = 1, T2 = 2, T3 = 3, T4 = 4; N stage was scored as N0 = 0, N1 = 1, N2 = 2, N3 = 3; TNM stage was scored as I = 1, II = 2, III = 3. The statistical analysis was carried out based on these definitions.

### 3.9 Percentages of patients with miR‐17‐5p, miR‐18a, miR‐20a, and miR‐210 high expression

Among the candidate miRNAs, high expressions of 4 miRNAs including miR‐17‐5p, miR‐18a, miR‐20a as well as miR‐210 were independent predictive factors for worse DFS and OS in GC patients. According to the expressions of these 4 miRNAs, the number of patients with ≥1 miRNA high expression, ≥2 miRNAs high expressions, ≥3 miRNAs high expressions, and 4 miRNAs high expressions were 298 (89.5%), 205 (61.6%), 118 (35.4%), and 43 (12.9%), respectively (Table 7).

**Table 7:** GC patients with miR‐17‐5p, miR‐18a, miR‐20a, and miR‐210 high expression

Parameters	GC patients (n %) (N = 333)
≥1 miRNA high expression	298 (89.5)
≥2 miRNAs high expressions	205 (61.6)
≥3 miRNAs high expressions	118 (35.4)
4 miRNAs high expressions	43 (12.9)

GC, gastric cancer.

Data were presented as count (%).

### 3.10 Correlation of the combination of miR‐17‐5p, miR‐18a, miR‐20a, and miR‐210 expressions with accumulating DFS

**Figure 6:**
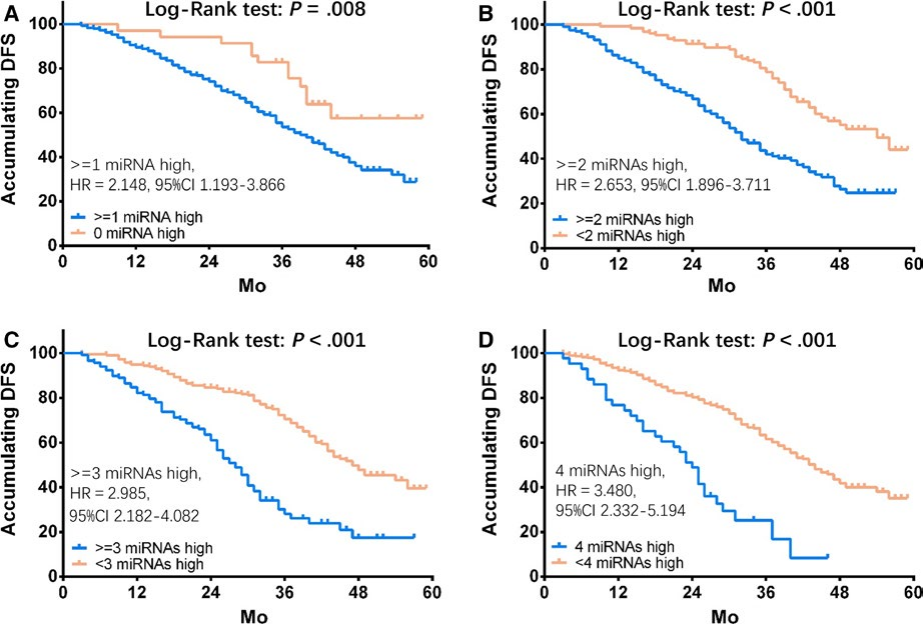
Correlation of DFS with ≥1 miRNA high expression, ≥2 miRNA high expression, ≥3 miRNA high expression, and 4 miRNAs high expression. DFS was shorter in GC patients with ≥1 miRNA high expression (vs 0 miRNA high expression) (A), ≥2 miRNA high expressions (vs <2 miRNA high expression) (B), ≥3 miRNA high expressions (vs <3 miRNA high expression) (C) as well as 4 miRNAs high expressions (vs 4 miRNAs high expression) (D). Moreover, as the numbers of miRNAs high expression increased, HR was numerically elevated, and a combination of these 4 miRNAs’ over‐expressions showed numerically best predictive value for poor DFS in GC patients. K‐M curves were applied to display DFS, and Log‐Rank test was used to compare DFS between groups. *P *<* *.05 was considered significant

As presented in Figure 6, DFS was worse in GC patients with ≥1 miRNA high expression (vs 0 miRNA high expression; Figure 6A, *P* = .008), ≥2 miRNAs high expressions (vs <2 miRNAs high expressions; Figure 6B, *P* < .001), ≥3 miRNAs high expressions (vs <3 miRNAs high expressions; Figure 6C, *P* < .001) as well as 4 miRNAs high expressions (vs <4 miRNAs high expressions; Figure 6D, *P* < .001). Furthermore, as the numbers of miRNAs high expression increased, hazard ratio (HR) was numerically raised, and the combination of these 4 miRNAs over‐expressions showed numerically best predictive value for poor DFS in GC patients.

### 3.11 Correlation of the combination of miR‐17‐5p, miR‐18a, miR‐20a, and miR‐210 expressions with accumulating OS

**Figure 7:**
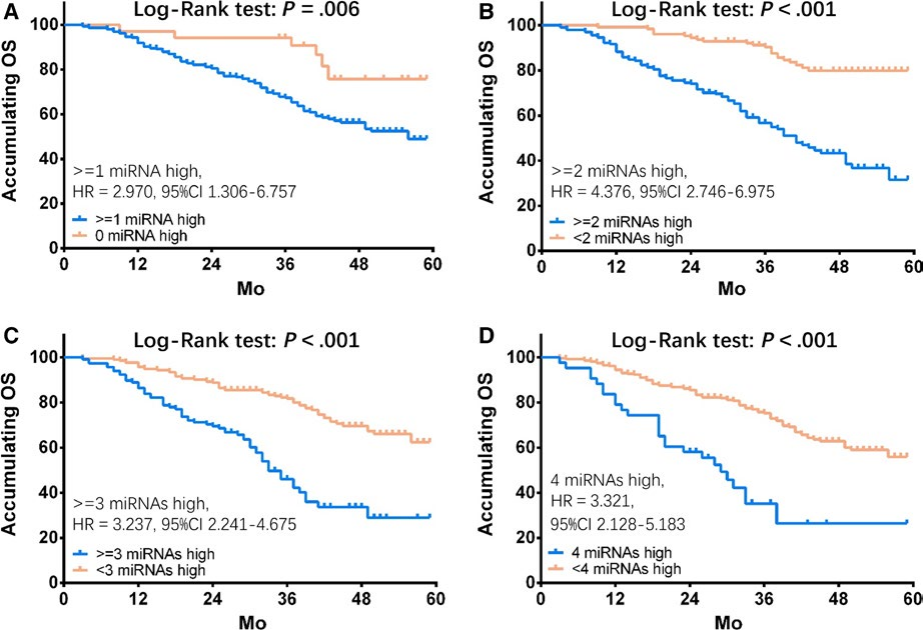
Comparison of OS with ≥1 miRNA high expression, ≥2 miRNA high expression, ≥3 miRNA high expression, and 4 miRNAs high expression. GC patients with ≥1 miRNA high expression (vs 0 miRNA high expression) (A), ≥2 miRNAs high expression (vs <2 miRNA high expression) (B), ≥3 miRNAs high expression (vs <3 miRNA high expression) (C), and 4 miRNAs high expression (vs 4 miRNAs high expression) (D) undertook worse OS. Furthermore, the highest HR value suggested that patients with ≥2 miRNAs high expression seemed to have the worst OS (B). K‐M curves were conducted to display OS, and Log‐Rank test was used to compare OS between groups. *P *<* *.05 was considered significant

GC patients with ≥1 miRNA high expression (vs 0 miRNAs high expression; Figure 7A, *P* = .006), ≥2 miRNAs high expressions (vs <2 miRNAs high expressions; Figure 7B, *P* < .001), ≥3 miRNAs high expressions (vs <3 miRNA high expressions; Figure 7C, *P* < .001), and 4 miRNAs high expressions (vs <4 miRNA high expressions; Figure 7D, *P* < .001) had worse OS. Moreover, the highest HR value was observed in Figure 7B (HR=4.376), suggesting that patients with ≥2 miRNAs high expressions were the best cut off for distinguishing OS in GC patients.

## 4 DISCUSSION

In this study, we found that: (1) Seven pro‐angiogenic miRNAs including let‐7f, miR‐17‐5p, miR‐18a, miR‐19b‐1, miR‐20a, miR‐210, and miR‐296 were elevated in GC patients compared with HCs; (2) Six pro‐angiogenic miRNAs including miR‐17‐5p, miR‐92a, miR‐210, miR‐20a, miR‐18a, and miR‐296 expressions were positively, while one pro‐angiogenic miRNA (miR‐130a) was negatively correlated with tumor malignancy degree in GC patients; (3) miR‐18a, miR‐20a, and miR‐210 disclosed good predictive values of GC risk; (4) high expressions of miR‐17‐5p, miR‐18a, miR‐20a, miR‐210, and miR‐19b‐1 were independent predictive factors for shorter DFS and OS, and a combination of these 4 miRNAs high expressions presented a good predictive value of poor DFS and OS in GC patients.

Tumor‐related angiogenesis has been proved to be a pre‐condition for growth and progression of solid tumors, and anti‐angiogenesis such as small molecule tyrosine kinase inhibitor and monoclonal antibodies are widely studied in cancer therapies since the past decades.^18^, ^19^ Furthermore, increasing studies have demonstrated that several miRNAs take part in the processes of angiogenesis and they play crucial roles in the progress or prognosis of cancers such as hepatocellular carcinoma, human colon cancer, and bladder cancer.^20^, ^21^, ^22^ For example, in a study conducted by Fang et al,^23^ miR‐93 represses integrin‐β8 expression, thereby allowing spreading of endothelial cells, and consequently promotes angiogenesis in glioma. It is also revealed that miR‐126 facilitates angiogenesis of GC through regulating vascular endothelial growth factor (VEGF) level, which is an important regulator in vasculogenesis.^18^ These studies indicate that miRNAs are involved in angiogenesis, and aberrant expressions of these miRNAs are associated with etiology and progression in cancers.^24^, ^25^, ^26^, ^27^, ^28^ However, the predictive value of angiogenesis‐related miRNAs in GC is rarely reported. Hence, we conducted this study to investigate the association of 14 angiogenesis‐related miRNAs with the risk of GC, and its correlation with diagnosis, clinicopathological features as well as survival profiles in GC patients.

Some studies have revealed that miRNAs have diagnostic value for cancers, while the diagnostic value of several pro‐angiogenic miRNAs for GC has rarely been systematically researched.^29^, ^30^, ^31^ In our study, we found that miR‐18a, miR‐20a, and miR‐210 had good predictive values for GC risk. The possible reasons might be that these pro‐angiogenic miRNAs might change the cells’ activity of gastric epithelial cells and enhance oncogenesis through stimulating some oncogenes, thereby leading to GC.^31^, ^32^, ^33^

Accumulating reports have explored the role of individual pro‐angiogenic miRNAs as biomarkers for the development and progression of various cancers. MiR‐17‐5p, belonging to the miR‐17‐92 cluster, has been found to be upregulated in various cancer tissues and correlated with advanced disease conditions including colorectal cancer, human breast cancer, lung cancer, pancreatic cancer, and so on, which promotes tumorigenesis, cancer cells proliferation, migration, and invasion via regulating P130, HMG box‐containing protein 1, TGFβ‐2 receptors, and other cancer‐related genes.^34^, ^35^, ^36^, ^37^ MiR‐18a, another pro‐angiogenic miRNA, is also discovered to be increased in several cancers including breast cancer, esophageal squamous cell carcinoma, and colorectal carcinoma, and it induces cancer cells’ proliferation, invasion, and autophagy through regulating PTEN‐PI3K‐AKT‐mTOR signaling axis and Dicer.^32^, ^38^, ^39^ miR‐20a is also regarded as an oncogene in several cancers such as cervical cancer, non‐small cell lung cancer, and anaplastic thyroid cancer, which raised cancer cells’ proliferation, invasion, and spheroid formation (stem cell ability) via targeting multiple genes including CYLD, ATG7, TIMP2, and LIMK1.^40^, ^41^, ^42^, ^43^ In addition, miR‐210, which leads to angiogenesis through stimulating VEGF production, is also discovered to be increased in several cancers, which correlates with higher pathological grade and elevated TNM stages, and it acts as an carcinogenic miRNA by stimulating cancer cells’ proliferation, migration, and invasion.^17^, ^44^, ^45^, ^46^, ^47^, ^48^ These studies imply that pro‐angiogenic miRNAs promote tumor development and progression, which serve as biomarkers of cancer risk and monitoring of malignancy degree in various cancers.

As in GC, a few studies also disclose that several pro‐angiogenic miRNAs are upregulated in GC tissues and associated with elevated disease stages. MiR‐17‐5p, as one of the frequently studied pro‐angiogenic miRNAs, is revealed to be elevated in cancer tissues and serum of GC patients and correlates with poor differentiation as well as higher TNM stage, which could promote GC cells’ proliferation and migration via several ways including posttranscriptional modulation of p21 and TP53INP1, transforming growth factor‐β receptor 2, and so on.^34^, ^49^, ^50^, ^51^ Another pro‐angiogenic miRNA (miR‐18a) is also observed to be raised in GC tissues compared with normal gastric tissues and is significantly correlated with higher pathological grade as well as lymph node status.^52^, ^53^ As with miR‐20a, it has been reported to be overexpressed in GC tissues and cells and positively correlates with tumor size, infiltration, and clinical grade, and it enhanced GC cells’ proliferation, migration, and invasion by regulating FBXO31, EGR2.^54^, ^55^, ^56^ In addition, the higher level of tissue miR‐210 expression in GC is also revealed in various studies, which illustrate that it correlates with larger tumor size, deeper invasion, higher possibility of distant metastasis as well as TNM stage.^57^, ^58^, ^59^ These studies suggest that pro‐angiogenic miRNAs also play critical roles in GC development and progression, and their expressions are increased in GC tissues and correlate with higher disease severity. However, these previous studies mainly investigate the expressions of pro‐angiogenic miRNAs in cancer tissues instead of blood samples, and each research only explores the expression of individual pro‐angiogenic miRNA in GC patients. In this present study, we found that 7 circulating pro‐angiogenic miRNAs including let‐7f, miR‐17‐5p, miR‐18a, miR‐19b‐1, miR‐20a, miR‐210, and miR‐296 were elevated in GC patients compared with HCs, and 6 pro‐angiogenic miRNAs including miR‐17‐5p, miR‐92a, miR‐210, miR‐20a, miR‐18a, and miR‐296 expressions were positively while one pro‐angiogenic miRNA (miR‐130a) was negatively correlated with tumor malignancy degree in GC patients. The results indicated that these circulating pro‐angiogenic miRNAs might be potential biomarkers for GC risk and indicators for malignancy degree in GC patients. The possible explanations were as follows: these pro‐angiogenic miRNAs induced carcinogenic transformation of normal gastric cells and promoted GC cells’ proliferation, migration, and invasion via regulating multiple oncogenous genes and related pathway, thus their expressions were upregulated and correlated with higher tumor stages in GC patients.

Accumulating studies also disclose that pro‐angiogenic miRNAs are present with good value in predicting prognosis in various cancers. Tumor tissue miR‐17‐5p high expression is illustrated to correlate with worse OS in nasopharyngeal carcinoma patients and colorectal cancer patients, and is an independent risk factor for DFS and OS in hepatocellular carcinoma patients.^33^, ^35^, ^60^ And tumor tissue miR‐18a high expression correlates with unfavorable OS in ovarian cancer patients and hepatocellular carcinoma patients.^61^, ^62^ The upregulation of tumor tissue miR‐20a is also observed to be associated with shorter OS in prostate cancer patients and gallbladder carcinoma patients.^63^, ^64^ In addition, tumor tissue miR‐210 high expression is illuminated to be correlated with worse DFS and OS in hepatocellular carcinoma patients, clear cell renal cell carcinoma patients, breast cancer patients, and so on.^47^, ^65^, ^66^, ^67^ And some meta‐analysis studies further demonstrate the prognostic role of miR‐210 in cancers.^47^, ^66^ As in GC patients, some reports also reveal that pro‐angiogenic miRNAs in tumor tissue correlate with unsatisfied prognosis such as miR‐17‐5p, miR‐20a, and miR‐210.^49^, ^68^ However, most of these previous investigations elucidate the effects of pro‐angiogenic miRNAs on prognosis in cancers based on their expressions in tumor tissues, while the tumor tissue samples are difficult to be obtain in many patients, particularly in older patients or terminal cancer patients who could not undergo surgery and unavailable to receive biopsy due to its invasiveness. Thus, the exploration of novel and feasible biomarkers for prognosis is essential for improving outcomes in GC patients. Currently, growing evidences have disclosed that the expressions of miRNAs originating from tumor tissues are detectable in blood samples by dissociation of cancer cells or transformation via exosome in cancer patients, and several miRNAs expressions in blood are shown to be correlated with the risk and clinicopathological features of various cancers.^69^, ^70^, ^71^ In this present study, we found that high expressions of 4 circulating pro‐angiogenic miRNAs including miR‐17‐5p, miR‐18a, miR‐20a, and miR‐210 were independent predictive factors for worse DFS and OS in GC patients; furthermore, the combination of these 4 circulating pro‐angiogenic miRNAs presented even better prognostic value for prognosis than individual one in GC patients. To our knowledge, this was the first report to integrate various circulating pro‐angiogenic miRNAs as the prognostic indicator in GC patients. The independent prognostic value of these circulating pro‐angiogenic miRNAs might result from: (1) These pro‐angiogenic miRNAs promoted angiogenesis to offer more blood supply for tumor growth by regulating target molecules or signaling pathways, such as HIF, Ephrin‐A3 (EFNA3), and tyrosine phosphatase PTP1B, thereby leading to severe disease degrees and increasing recurrence in GC patients, which subsequently resulted in worse prognosis; (2) These pro‐angiogenic miRNAs promoted GC cells’ proliferation, migration, and invasion while inhibited cells’ apoptosis through regulating multiple oncogenes and cancer‐related pathways, which greatly improved the GC malignancy degree and led to unsatisfied prognosis in GC patients; (3) These pro‐angiogenic miRNAs enhanced GC cells’ resistance to several treatment drugs and radiotherapy, which deteriorated prognosis in GC patients.

Our study still had some limitations: (1) Sample size of this study was relatively small, which reduced statistical power and increased the influence of extreme values; (2) GC patients were recruited in a single cohort center, which might cause selection bias; (3) No GC patient with TNM stage IV was included in this study, thus the role of these 14 pro‐angiogenic miRNAs in GC was not explored.

In conclusion, circulating pro‐angiogenic miRNAs could serve as novel noninvasive biomarkers for disease risk and malignancy degree, and miR‐17‐5p, miR‐18a, miR‐20a, and miR‐210 are independent factors predicting poor prognosis in GC patients.

## CONFLICTS OF INTEREST

No conflicts of interest to disclose.

## ETHICS

This present study was approved by the Ethics Committee of Guangdong General Hospital Zhuhai Hospital, Zhuhai Golden Bay Central Hospital and conducted according to the Helsinki declaration. Each GC patient and HC provided written informed consent before being included in the study.
