# Supplementary material for: The correlation of circulating pro‐angiogenic miRNAs’ expressions with disease risk, clinicopathological features, and survival profiles in gastric cancer
Source: Cancer Med. 2018 Jul 12;7(8):3773–91. doi: 10.1002/cam4.1618 (PMC6089172; doi:10.1002/cam4.1618)
Supplement: Supplementary file 1 [file CAM4-7-3773-s001.docx]

**Supplementary Table 1.** Summary of miRNAs expressions, diagnostic value and prognostic value

|  | Expression  (Wilcoxon rank sum test) | | Diagnostic value  (ROC) | | Prognostic value  (Multivariate Cox’s proportional hazard regression) | | | |
| --- | --- | --- | --- | --- | --- | --- | --- | --- |
| miRNAs | HCs | GC patients | AUC | 95%CI | DFS (high vs low) | | OS (high vs low) | |
|  |  |  |  |  | P value | HR | P value | HR |
| let-7b | NS | NS | 0.541 | 0.482-0.600 | 0.957 | 0.991 | 0.461 | 0.859 |
| let-7f | low | high | 0.573 | 0.516-0.629 | 0.447 | 0.880 | 0.268 | 0.798 |
| miR-17-5p | low | high | 0.594 | 0.538-0.650 | **<0.001** | 1.930 | **<0.001** | 2.308 |
| miR-17-3p | NS | NS | 0.528 | 0.469-0.586 | 0.112 | 1.307 | 0.210 | 1.294 |
| miR-18a | low | high | 0.632 | 0.577-0.686 | **<0.001** | 1.876 | **0.001** | 1.977 |
| miR-19a | NS | NS | 0.535 | 0.474-0.595 | 0.964 | 0.993 | 0.907 | 0.977 |
| miR-19b-1 | low | high | 0.561 | 0.502-0.621 | **0.045** | 1.421 | 0.204 | 1.306 |
| miR-20a | low | high | 0.608 | 0.550-0.666 | **0.003** | 1.679 | **0.001** | 2.067 |
| miR-92a | NS | NS | 0.536 | 0.479-0.594 | 0.073 | 1.353 | 0.182 | 1.309 |
| miR-126 | NS | NS | 0.554 | 0.494-0.615 | 0.513 | 0.893 | 0.814 | 0.953 |
| miR-130a | NS | NS | 0.537 | 0.478-0.595 | 0.056 | 1.376 | 0.058 | 1.493 |
| miR-210 | low | high | 0.608 | 0.548-0.667 | **0.015** | 1.531 | **0.024** | 1.577 |
| miR-296 | low | high | 0.564 | 0.503-0.626 | 0.391 | 1.152 | 0.252 | 1.263 |
| miR-378 | NS | NS | 0.541 | 0.481-0.600 | 0.902 | 1.020 | 0.561 | 1.121 |

HCs, health controls; GC, gastric cancer; NS, no significance; ROC, Receiver Operating Characteristic curve; AUC, area under curve; CI, confidence interval; DFS, disease free survival; OS, overall survival; HR, hazard ratio.
